# Living with type 1 diabetes in Neno, Malawi: a qualitative study of self-management and experiences in care

**DOI:** 10.1186/s12913-023-09519-z

**Published:** 2023-06-08

**Authors:** Laura Drown, Alma J Adler, Leah N. Schwartz, Junious Sichali, Francis Valeta, Chantelle Boudreaux, Celina Trujillo, Todd Ruderman, Gene Bukhman

**Affiliations:** 1 Center for Integration Science, Division of Global Health Equity, Department of Medicine, Brigham and Women’s Hospital, 75 Francis St, Boston, MA 02115 USA; 2 Harvard Medical School, Boston, MA USA; 3 Partners In Health/Abwenzi Pa Za Umoyo, Neno, Malawi; 4 Partners In Health, Boston, MA USA; 5 Program in Global Noncommunicable Disease and Social Change, Department of Global Health and Social Medicine, Harvard Medical School, Boston, MA USA

**Keywords:** Type 1 diabetes, Malawi, Qualitative study

## Abstract

**Background:**

The prevalence of type 1 diabetes (T1D) is increasing in low-income countries including Malawi. In this setting, care is frequently impacted by challenges in diagnosis and management. Access to high-quality T1D care remains limited in Malawi, with fairly low availability and high cost of insulin and other supplies and diagnostics, lack of T1D knowledge, and absence of readily accessible guidelines. In the Neno district, Partners In Health established advanced care clinics at district hospitals to provide comprehensive, free care for T1D and other noncommunicable diseases. Prior to this study, experiences in care for people living with T1D (PLWT1D) at these clinics remained unexplored. Here we examine the impact of living with T1D, knowledge and self-management of, and facilitators and barriers to T1D care in Neno District, Malawi.

**Methods:**

We conducted a qualitative study utilizing behavior change theory that consisted of twenty-three semi-structured interviews conducted in Neno, Malawi in January 2021 with PLWT1D, their families, providers, and civil society members to explore the psychosocial and economic impact of living with T1D, T1D knowledge and self-management, and facilitators and barriers to accessing care. Interviews were analyzed thematically using a deductive approach.

**Results:**

We found that PLWT1D had good knowledge and practice of self-management activities for T1D. Key facilitators to care identified by informants included extensive patient education and availability and provision of free insulin and supplies. Significant barriers included distance from health facilities, food insecurity, and low literacy/numeracy. Informants described T1D as having a notable psychosocial and economic impact on PWLT1D and their families, notably worrying about having a lifelong condition, high transportation costs, and reduced working ability. While home visits and transport refunds helped facilitate access to the clinic, informants reported the refunds as inadequate given high transport costs faced by patients.

**Conclusions:**

T1D was found to have a significant impact on PLWT1D and their families. Our findings represent important areas of consideration in design and implementation of effective programs for treating PLWT1D in resource-limited settings. Facilitators to care identified by informants may be applicable and beneficial in similar settings, while persisting barriers represent areas for continued improvement in Neno.

**Supplementary Information:**

The online version contains supplementary material available at 10.1186/s12913-023-09519-z.

## Background

Type 1 diabetes (T1D), a severe chronic autoimmune disease in which the pancreas produces little or no insulin, is among one of many non-communicable diseases (NCDs) representing a growing proportion of the burden of disease in sub-Saharan Africa (SSA) [1, 2] While epidemiological data for T1D in the region is lacking, the International Diabetes Federation (IDF) reports diabetes to be on the rise in Malawi. The IDF estimated that 486,000 adults in Malawi were living with diabetes of both types in 2021 of which approximately 58% are likely to be undiagnosed and therefore still untreated [3]. The 2017 Malawi National STEPwise Survey for Non-Communicable Diseases Risk Factors reported an estimated prevalence of 1.4%, representing about 247,000 individuals expected to have diabetes [4]. Importantly, it notes that 96% of respondents reported never having their blood glucose measured and the need for increased screening.

Despite the growing burden of diabetes in Malawi, access to high-quality care remains limited. For people living with type 1 diabetes (PLWT1D), daily insulin administration is necessary to manage blood glucose (BG) levels. However, access to insulin and other essential supplies (e.g., syringes, BG monitoring devices) remains a barrier to proper management of and survival with T1D. Lack of access to insulin is the main cause of mortality for children with T1D globally and for those in SSA, life expectancy after diagnosis can be as low as one year [5]. A recent study found that only 58% of public district hospitals in Malawi had insulin available and a similar proportion (56%) had glucose monitoring equipment available [6]. A study of diabetes care in a rural district of the country similarly found shortages in medicine and reagents for tests and other issues with care, including lack of knowledge of the disease in both PLWT1D and providers and absence of diabetes guidelines [7]. Even if available at health facilities, cost of insulin may be a barrier to care. On average, a yearly supply of insulin in Malawi costs 43% of the annual median income in the country [8].

Partners In Health (PIH), a non-governmental organization (NGO) known in Malawi as Abwenzi Pa Za Umoyo (APZU), established the country’s first advanced NCD clinics at two district level hospitals in the rural Neno district in 2018 [9, 10]. These clinics were preceded in 2011 by the integrated chronic care clinic, a novel approach integrating NCD and HIV services at the same clinic [11]. The advanced care clinics provide comprehensive, integrated care for severe NCDs, such as T1D, through the WHO Package of Essential Noncommunicable Disease Interventions – Plus (PEN-Plus) strategy. This allows for closer monitoring and additional services for patients with these conditions [12]. Until now, experiences in care for PLWT1D enrolled in this clinic have not been explored. Literature on T1D care in Malawi and in SSA more broadly remains lacking. Our team previously conducted a qualitative study on T1D care at PIH-supported PEN-Plus clinics in Liberia. Here, we describe a similar study in Malawi to further build on this body of evidence [13]. In this study, we examine the impact of living with T1D, knowledge of the disease and self-management, and facilitators and barriers to T1D care at two clinics in Neno.

## Methods

### Framework

Our study used Michie and colleagues’ Behaviour Change Wheel (BCW) framework to inform the development of semi-structured qualitative interview guides exploring the experiences of PLWT1D and health care providers with diabetes management [14–16]. The BCW framework describes three distinct categories that interact to influence behavior, namely capability, opportunity, and motivation. In our study, we specifically focused on the first two categories—capability and opportunity— since we felt the scope of our organization’s work was best suited to address these facets of diabetes management. Together with our team’s clinical and programmatic experience, this framework guided development of qualitative interview tools (Appendix 1) that aimed to explore and understand the (1) perception of all involved individuals on individual and health systems level barriers and facilitators for accessing T1D care, (2) experiences of PLWT1D and their families with self-management of T1D, (3) psychosocial and economic impact of living with T1D on PLWT1D and their families, (4) PLWT1D’s and families’ knowledge of T1D management and treatment [16].

### Data collection

Semi-structured qualitative interviews were conducted at two PEN-Plus clinics in first level hospitals in the Neno district or via telephone (civil society members) as necessary by a trained male qualitative researcher (JS) with no clinical duties or previous connection to the study sites or informants. PIH/APZU works with the Ministry of Health to provide periodic hemoglobin A1c, diabetes education, and free provision of insulin and other supplies for PLWT1D at these two clinics. Study informants included (1) PLWT1D receiving care at PEN-Plus clinics in Neno, (2) family members of PLWT1D receiving care at these clinics, (3) health care providers of T1D care in Neno, and (4) civil society members working in the area of T1D in Malawi (Table 1). Interview question topics varied by participant type as shown in Table 1. Informants were selected based on T1D diagnosis or role in T1D care, basic characteristics, availability for interviewing, and willingness to participate in the study. The NCD nurse at the clinics of interest invited potential informants to be interviewed during their regular clinic visits. No potential informants declined to participate. All informants provided verbal or written consent prior to the start of the interviews, which were conducted privately. Interviews were carried out in Chichewa, the local language of this area, using standardized semi-structured interview tools and were audio recorded. Interviews lasted approximately one hour in duration. Interview guides were developed by co-authors building on previous research conducted by the authors [13, 17, 18]. We conducted interviews until thematic saturation was reached, considering each informant group independently. This methodology follows that used in our previous qualitative study in Liberia and utilizes similar interview tools [13].

**Table 1:** Informant characteristics and interview question topics

Informant type	Interview topics
PLWT1D (n = 8)	Knowledge of diabetes and diagnosis Management Healthcare experiences and recommendations Facilitators and barriers to care Adherence Self-management Effect on daily and community life Catastrophic costs Lifestyle choices
Family (n = 4)	Burden of illness on family Management Facilitators and barriers to care Everyday life changes due to T1D Costs
Provider (n = 9)	Knowledge of diabetes and diagnosis Experience and tasks Care provision and coordination Facilitators and barriers Relationship with patients Provision of social support Screening for mental health Health systems level areas for improvement Training
Civil society member (n = 2)	Role of civil society Services provided and recipients Facilitators and barriers to providing services Challenges in access Availability of social support for families

### Data analysis

All interviews were transcribed and translated from audio recordings then coded in Dedoose version 8.3.35 [19]. Dedoose was used by a co-author (LD) to apply codes to interview transcripts from a codebook previously utilized in this group’s previous similar study in Liberia and modified as appropriate [13]. Analysis was comprised of two iterative steps, beginning with a deductive, *a priori* thematic analysis of one initial interview and later expanding the coding structure to include additional themes that emerged from the first step (Appendix 2). We used the revised coding structure to recode the initial interview and all subsequent interviews as needed. Coded transcript excerpts were subsequently exported from Dedoose and reviewed to identify the most common themes across all interviews.

## Results

We conducted qualitative interviews with twenty-three informants, primarily PLWT1D (34.8%) and providers (39.1%) (Table 1). The mean age of PLWT1D interviewed was 31.8 years and of those for whom sex was reported, the majority were male (66.6%).

### Diagnosis

According to providers, T1D is typically diagnosed through BG testing, particularly for patients presenting with characteristic symptoms. PLWT1D, families, and providers described a wide range of conditions in which new T1D patients present at diagnosis. PLWT1D and their families reported experiencing symptoms such as headache, stomach pain, weight loss, polyuria, polydipsia (excessive drinking of water), polyphagia (excessive hunger), and weakness prior to diagnosis. According to providers, some present in later stages of the disease with complications, even arriving at the hospital unconscious.

***Provider 4*** *So a patient will come in the ward or OPD and say that I am having frequent urination, am frequently taking a lot of water, am always thirsty, sometimes I feel dizzy, sometimes I do sweat, if its somebody of age, it is a bit different from the one with age 10 and below. They may not say I am urinating frequently but the guardian will say the child is passing frequent urine. So if we come across those complaints, we check their sugars and we find that the sugars are high.*

Providers noted that patient age is an important characteristic considered in diagnosis of T1D in the absence of laboratory tests like antibodies, C-peptide, and ketones. After a patient is determined to have diabetes, classification of diabetes type is based on his or her age, with a type 1 diagnosis assigned to younger patients. Co-authors working in Neno elaborated on the process of differentiating between type 1 and 2 diabetes. They explained that for patients between the ages of thirty and forty-five, providers consider additional factors such as body mass index, comorbidities, and presenting symptoms. Following administration of insulin and once BG levels are stable, some inpatients with diabetes are also given metformin while still being monitored in the hospital to determine if oral diabetes medications used to treat type 2 diabetes are effective.

Some PLWT1D explained the difficulties they faced in obtaining a T1D diagnosis. Although screening takes place in communities and facilities, they described not being diagnosed at health centers after developing symptoms. One patient was referred to the district hospital after her weight loss remained unresolved at the health center. They remained in inpatient care for a month before finally receiving a T1D diagnosis.

***PLWT1D 1*** *At first I was losing weight and then I decided to go to [health center] where they gave me flour because they thought that I was malnourished. However, this did not work because I continued losing weight. They conducted tests but could not discover what I was suffering from but this time they started giving me nutritional stuff/foods (chiponde) so that I could gain weight but still there was no improvement. This was the time they decided to refer me to [District Hospital 1). I was admitted at [District Hospital 1] for one month and they could not get the diagnosis but by the time I was about to be discharged they discovered that I was suffering from Type 1 diabetes and I started receiving treatment that same day.*

The family of another PLWT1D described visiting other health facilities, including private clinics, without diagnosis. After this PLWT1D collapsed at one of these facilities, a doctor arranged ambulance transport to the district hospital where they received a diagnosis of T1D.

These experiences of PLWT1D and their families align with systems-levels barriers to care identified by providers and civil society members. A civil society member described a lack of attention to T1D paid by the national government and resulting barriers to care in the country.

***Civil society 1*** *The government does not take care of diabetic patients. There were a lot of challenges for diabetic patients to receive care… The problem is that the government at first was not giving attention to diabetic patients.*

Informants described overall diagnostic capacity at health facilities in Malawi as low, particularly at the lower health system levels. Lack of diagnostic equipment and awareness, particularly in providers not trained in this area, contribute to underdiagnosis of T1D and diagnosis at later stages with complications.

***Provider 7*** *I think because we have under-diagnosed it or not recognizing it, that is the reason it can escape in our system. However, it remains a burden because most patients they come to our facilities at a later stage when, for example, they have complications already. So to reverse it, you have to deal with the complication at hand, for example, some have chronic wounds which need amputation. They didn’t recognize that they have a problem or they have diabetes but they come in with it at a very late stage.*

Some providers related this to the lack of priority and attention given to T1D and other NCDs in Malawi compared to infectious diseases. Many PLWT1D and their families confirmed that they did not have any knowledge of T1D prior to initial diagnosis.

### Management

PLWT1D visit the clinic monthly. Providers described a wide range of activities conducted on routine visits, including lab testing, review of logbooks of home glucose testing, medication management and dispensation, patient education, and screening for other conditions.

Provision of free insulin and supplies was commonly mentioned as a key facilitator to care. All PLWT1D seen at these clinics receive free insulin and other supplies including syringes, home glucometers, and test strips. At clinic visits, PLWT1D reported being assisted quickly, few stockouts, and receiving sufficient supplies until their next scheduled visit. They report no need to ration or skip insulin doses unless they miss a clinic visit. Providers echoed these statements, reporting good availability of insulin and supplies at these facilities. Similarly, distribution of transport refunds to PLWT1D facilitates access to these free services for those living outside of the catchment areas. While PLWT1D, families, and providers identified transport refunds as a facilitator to care, some described them as inadequate given the long travel distances faced by many PLWT1D.

***Provider 4*** *Of now they are providing transport to these patients to and from but most of the time I see patients complaining that what they are being given is not enough. They are being given two-way transport but for that two way to them, it’s more of one way transport so they don’t have money and they sacrifice themselves to walk home and use that money for food.*

Patients were given the opportunity to provide feedback on T1D services in Neno. Overall, PLWT1D and their families expressed that they felt satisfied with the T1D care they received.

***Provider 4*** *In my personal opinion I feel like it is very good. They really help us a lot.*

In contrast to the comprehensive care provided at Neno, informants described T1D diagnosis and care outside of the district as scarce, with limited access to insulin or diagnostics at most government facilities.

***Provider 7*** *I think, from government perspective, I think there is one thing that has brought challenges in terms of supply. Mostly you see that facilities don’t have right equipment to diagnose diabetes, especially type 1, when it presents early.*

#### Education

Providers described patient education as a key component of every clinic visit and discussed conducting continuous education covering a wide range of topics, including usage and storage of insulin, prevention and treatment of hypo- and hyperglycemia, diet, exercise, foot care, injection-site care, glucometer use, and prevention of injuries. They noted the positive impact of this education on adherence and outcomes in PLWT1D. While newly diagnosed PLWT1D or those with low literacy levels can initially struggle to understand self-management practices, continued education results in improved diabetes self-management practices and, thus, better outcomes for most PLWT1D.

***Provider 2*** *There are many things that work well depending on the stages and how long the client has been in care. For beginners, they do struggle to understand how to use drugs, where to inject, making trips to the facility, how to store drugs. But as time goes, most of them understand the whole protocol and begin to adhere to whatever we tell them to do. This has resulted into having more clients who are stable.*

Though education is primarily provided by nurses and clinical officers at clinic visits, informants also discussed education conducted by pharmacists and community health workers (CHWs). CHWs were reported to focus primarily on T1D symptoms and appropriate care-seeking, while pharmacists focus on insulin use and storage.

According to family members, inclusion in education enabled them to assist patients in self-management and encourage adherence. Providers noted that inclusion of parents or partners is important for PLWT1D, as they are often children who need extra help for self-management activities. Family members of younger patients described accompanying PLWT1D to clinic visits, checking BG levels, injecting insulin, and performing other self-management activities for them in instances of hyper- or hypoglycemia. Older PLWT1D typically reported performing daily self-management activities independently but described family members providing support as needed when they were feeling ill or weak. For PLWT1D reporting ability to conduct self-management activities, caregivers provided emotional support and encouragement.

***Provider 2*** *Usually one of the guardians has to know so that they can remind the client if they miss something, especially how to draw medication, storage and injections. They have to be supportive. Sometimes the client may say no, today am tired and am not doing it, the guardian should be able to encourage them to still do it.*

#### Home visits and support

While all PLWT1D participating in this study reported visiting the clinic regularly, providers discussed conducting home visits for patients who are unable to attend clinic visits due to mobility or other issues. Home visits are also conducted for PLWT1D receiving care at the clinic so providers can observe self-management practices. Providers reported being in regular contact with PLWT1D via telephone to provide additional support and encourage adherence.

***Provider 1*** *Most of the T1D [patients] have phones and the nurse who is the focal person on our side interacts often and, if there is something requiring my support, I come in quickly. If a patient has something to communicate to us, they call the nurse who reaches out to me with the information and if our part we also want to talk to the patient, then the nurse just calls.*

A network of CHWs provide additional at-home support to PLWT1D. Providers described the role of CHWs in visiting PLWT1D in their homes, providing reminders and reinforcements of self-management education, and encouraging care-seeking at the hospital. However, most PLWT1D stated that CHWs either did not visit their homes or did not visit on a regular basis. Some described how CHWs provide support and encouragement regarding their condition.

***PLWT1D 4*** *I feel like he helps me a lot because he encourages me on what I am supposed to remain healthy, especially management of my condition by following health workers’ advice. When I am down, he encourages me a lot.*

#### Self-management

Providers noted that all PLWT1D enrolled in the clinic initiate insulin following diagnosis. PLWT1D described regularly using two types of insulin – intermediate-acting (Lente) and short-acting (Actrapid). Nearly all patients inject insulin twice a day, though two reported that they take insulin three times a day. Overall, PLWT1D and their families reported taking insulin doses as prescribed.

They reported always using a full dose of insulin, facilitated by good availability of free insulin at the clinics. Few PLWT1D reported ever missing doses of insulin and explained that these rare occurrences were related to food insecurity and work. One reported sometimes skipping insulin injections because he had not eaten anything and could become unconscious after injecting. Another described sometimes having to spend a night in another district on business and missing an evening dose in those instances.

Nearly all PLWT1D reported testing their BG levels once a day using a clinic-provided home glucometer and recording results in a logbook. They discussed using these results to manage their condition, responding to episodes of hyper- and hypoglycemia according to education received at the clinic. They typically consume a spoonful of sugar, sweet foods, or juice when experiencing low BG. Management of hyperglycemia often consists of exercise, doing manual labor, or drinking water. A few PLWT1D reported injecting insulin when their BG is high.

Both PLWT1D and their families discussed recognizing symptoms of abnormal BG levels. They noted fatigue, sweating, and shivering as symptoms of hypoglycemia and headache, vision problems, red palms, and body pains as those of hyperglycemia. PLWT1D explained that these symptoms prompt them to check their BG and manage accordingly.

Providers identified additional barriers affecting adherence to treatment, including socioeconomic status and low literacy/numeracy level. Socioeconomic status may impact the ability to reach the clinic due to the high transport costs for many patients. Consistency with management activities, particularly injection of the correct prescribed dose of insulin on a regular basis, is a challenge for some PLWT1D. Though comprehensive and continuous education may mitigate this challenge to self-management for most PLWT1D, low literacy or numeracy level can be an initial or even long-term challenge to certain aspects of self-management for others.

***Provider 4*** *The problem is always from the patient. That is about literacy, sometimes whenever we are discussing about how to draw the medication and when it comes to figures, these things become a problem. And for patients who have problems with eyesight, as a complication of [diabetes], it becomes a challenge for them to draw the accurate figures of insulin, the dosage. So if they don’t have a guardian at that time, it’s always difficult. You may find that today they draw a different amount of insulin, and tomorrow another dose. And for those that are illiterate, if you ask them how much do you give yourself, today they tell you another figure and tomorrow another figure. So it’s quite difficult for them.*

Some PLWT1D reported use of traditional medicine. One PLWT1D was advised by several people in their community to use herbs to treat their T1D, but refused. Another briefly tried medicine from a traditional healer but discontinued fearing complications after his BG levels continued to rise. A provider explained that use of country medicine from herbalists may affect management of T1D.

***Provider 3*** *Neno is one of the districts where the residents believe much in magic so usually when you tell them this is diabetes, it starts like this, it doesn’t have medication, its incurable, they feel like no, may be somebody has bewitched them. So maybe they have to consult any herbalist for a better medication. As such sometimes they tend to default our programs.*

#### Diet

Nearly all informants discussed modification of diets following diagnosis with T1D. Many PLWT1D mentioned avoiding very sweet foods and beverages, as well as salty and oily foods that affect BG levels. Providers noted that while diet advice is part of diabetes education, it is often difficult for patients to follow this guidance and adhere to the recommended diet due to PLWT1D’s socioeconomic status, the cost of food, and presence of food insecurity. One explained that the diet recommended to PLWT1D is not affordable to most families and should be more realistic to this setting.

***Provider 2*** *Asking a poor family to ensure that their meals should include two slices of bread, two lemons, fried chips and chicken etc. is not feasible because the majority of Malawian population is poor. In reality we should be stressing on diet that someone can afford and not on things that they can merely dream of.*

Food insecurity was a significant barrier reported by patients and providers. While insulin should be injected after meals, lack of food forces some PLWT1D to either inject it on an empty stomach or skip doses.

***Provider 8*** *With our financial status and education level, it is not easy for our clients to adhere to this like I have taken my shot at this hour and then I have to eat after 30 min. So they will take a shot but they don’t have the food to take immediately. Or they will take the shot and not take the food like the whole day but take the food in the evening. Some have even admitted saying I have had hunger problems at home, as a result I stopped giving myself insulin because I was very hungry when I get a shot*.

Patients confirmed not being able to follow dietary recommendations given by providers because of the cost of food.

***PLWT1D 5*** *I do eat but not as I was advised by health workers because we cannot manage to have them. For example, they told me to ensure that each morning I should eat before taking insulin shots. This is not possible with me because my parents cannot afford breakfast for the family every morning. This is very difficult and sometimes I do not eat anything due to food unavailability.*

### Costs

Though services, insulin, and supplies are provided free of charge to PLWT1D patients at Neno facilities, numerous informants discussed additional costs to PLWT1D and their families. Cost of transport was by far the most frequently mentioned economic impact. However, several informants discussed how T1D affected their ability to work and earn money. For some PLWT1D, this condition and the resulting weakness has forced them to either sometimes miss work or reduce the amount of work they do.

***PLWT1D 3*** *I used to think that in future I should be independent after I work hard to support myself. But due to my condition I am not working as I am supposed to do because sometimes I am weak so I do not work properly and sometimes I even fail to work*.

Caregivers are also impacted, with some PLWT1D reporting that their family member or guardian sometimes misses work to care from them if they are sick.

A large majority of informants identified long distance from the hospital as a barrier to care. Most PLWT1D reported traveling to the hospital by motorcycle or on foot. The time PLWT1D spend traveling from home to the hospital ranged widely from 30 min to five hours. They typically pay between 3,000 ($3.86 USD) and 12,000 ($15.46 USD) Malawian kwacha for round trip transportation to the hospital. A transport refund of 3,400 kwacha ($4.38 USD) is provided to PLWT1D living outside of the hospital’s catchment area, but many informants expressed that this amount is not adequate to fully cover transport costs. One patient reported missing a clinic appointment because they did not have enough transport money, despite receiving transportation funds.

Discussion surrounding social support resources available to PLWT1D varied. While two PLWT1D reported once participating in a support group at the hospital a long time prior to the interview and another was aware of the group but had not yet participated, most PLWT1D were unaware of the existence of this type of support. Providers discussed linking PLWT1D experiencing food insecurity to APZU’s Program for Social and Economic Rights for food aid. However, ongoing aid for food insecurity was not mentioned by any PLWT1D or their families when asked about social support they received, despite describing the food insecurity they had experienced. PLWT1D and providers described a previous social support program in which PLWT1D received monthly cash transfers of 15,000 Kwacha (~$20 USD) for five months during the COVID-19 pandemic and expressed the need for additional cash transfers to support their basic needs.

### Psychosocial impacts

When asked about the impact of T1D on their mental health, some PLWT1D noted that they worry about their condition and now have a changed outlook on their futures.

***PLWT1D 3*** *I used to think that in future I should be independent after I work hard to support myself. But due to my condition I am not working as I am supposed to do because sometimes I am weak so I do not work properly and sometimes I even fail to work.*

*Interviewer: how do you look at your future?*

*Respondent: It is not good at all.*

Family members shared similar concerns, expressing worry over their loved one’s condition and the impact on their future.

***Family 1*** *Worries can’t fail to be there… The future might indeed be uncertain since he is often sick*.

Another PLWT1D related her worries about T1D to the lifelong insulin management required of the condition. However, others had contrasting views – they noted that they do not worry about having T1D because they have accepted their diagnosis and follow the advice of health workers.

***PLWT1D 2*** *The main issue is following instructions of healthcare workers especially on how to manage our conditions. If we follow whatever we are told by health workers, then everything will be fine especially regarding the proper use of insulin.*

Some PLWT1D explained that while T1D has changed their lives, particularly with regards to how they are able to work and take care of themselves independently, their lives have improved since being diagnosed. Informants reported improved health and quality of life after initiating treatment due to improvement of T1D symptoms and overall health. Though some PLWT1D still rely on their families to care for and support them, others reported now living more normally and being able to work.

***PLWT1D 1*** *A lot has changed in my life because at first I did not have good health like this. My life was tough because I was suffering a lot and lost weight but when I got diagnosed and initiated on treatment I have seen great improvement in my general health and I have even gained more weight than before I started treatment.*

## Discussion

### Key findings

We found that PLWT1D had high capability to manage their condition at home, including good knowledge and practice of self-management activities such as insulin injection and home glucose monitoring, likely because of extensive education received at clinic visits. Many factors of care provision specific to Neno improved the opportunity for patients to receive care and manage their condition, though barriers to opportunity remain as areas for improvement. Provision of free insulin and materials was also a significant facilitator to care for these individuals, who receive subsidized care at PIH-supported facilities. Transport funds distributed to PLWT1D living in certain areas and home visits by providers as well as CHWs additionally increase access to T1D care in Neno. In contrast, distance from the health facilities was reported to be a barrier by numerous informants. We found that food insecurity and low literacy also affected adherence to treatment. Informants described both psychosocial and economic impacts of T1D. They described worrying about their condition, particularly due to the lifelong management it requires, and viewing their future outlooks differently following diagnosis. While PLWT1D receive free care, insulin, and materials, they noted additional costs associated with their condition, such as high transportation costs and reduced ability to work.

In describing T1D care at PEN-Plus clinics in Neno, where NGO-supported services provide free and comprehensive care, informants highlighted contrasts with T1D care in Malawi more broadly. On a national level, informants reported low awareness of T1D in both the general population and providers, and low prioritization of this condition by the government. While informants noted provision of free insulin and materials as a key facilitator to PLWT1D, this aspect of care is unique to Neno. Literature suggests that elsewhere in Malawi, insulin is not reliably available or affordable to PLWT1D [6–8]. Distribution of free self-monitoring blood glucose devices to PLWT1D for daily use at home is another important and unique aspect of this program to aid patients in effective self-management and survival [20]. Ability to monitor blood glucose at home helps prevent severe hypoglycemia and facilitates adjustment of insulin dosages as needed.

We conducted a similar qualitative study of T1D in Liberia, which was the first study of its kind in a low-income country in SSA [13]. Both studies found T1D to have a major economic and psychosocial impact on PLWT1D and their families. Patient education was reported to be a key activity in care in both studies, contributing to increased patient knowledge and frequency of diabetes self-management behaviors. Our study in Liberia similarly found reliable provision of free services and materials as to be important systems-level facilitators to care. Both studies reported PLWT1D distance to the clinic, food insecurity, PLWT1D literacy/numeracy and lack of awareness and prioritization of T1D in the larger health system and community as significant barriers to care.

Unlike the Liberia study, this study identified use of traditional medicine to be a potential barrier to care for some PLWT1D, leading patients to default from clinic programs in favor of herbal remedies. Some facilitators were unique to the context of Neno district, including provision of transport refunds and existence of a CHW network offering support to PLWT1D in their homes. Financial support for transport was an important feature of Neno’s program that aided PLWT1D in adhering to a monthly visit schedule and procuring insulin in a timely manner. Though not all PLWT1D reported interacting with CHWs, the support they offer in facilitating care-seeking, adherence to self-management practices, and providing additional emotional support and encouragement was noted to be helpful. Since T1D is a lifelong condition requiring extensive self-management, this study demonstrates that existing CHW programs offer a valuable opportunity to support these PLWT1D in their own homes.

Other qualitative studies of insulin-treated diabetes and T1D in SSA report similar themes consistent with this study. They also identified barriers to care such as misdiagnosis, dietary challenges, skipped insulin doses due to food insecurity, and the significant financial and psychosocial burden of T1D [21–23].

### Strengths and limitations

This is one of the first studies to explore experiences living with T1D in rural sub-Saharan Africa. We conducted interviews with a wide range of informant types associated with T1D care in Neno district including providers, patients, families, policy makers and civil society members. Inclusion of these informant types in qualitative interviews allowed us to gain a comprehensive view of multiple facets in the experience of living with T1D in this district.

All PLWT1D and families interviewed as part of this study received care at PIH-supported facilities. Therefore, they receive subsidized care within an integrated service delivery model. Given the rather low availability of T1D care at the national level, these patients and their experiences in care are not representative of the broader population of PLWT1D in Malawi.

Another potential limitation of this study is the setting in which interviews were conducted. Many of these interviews took place at the clinics where PLWT1D were routinely receiving care. Therefore, PLWT1D may have been less likely to speak freely about their experiences in care than if interviews had been conducted in their homes. Similarly, providers’ responses about care provision may have been impacted since they were interviewed at their place of work.

### Future implications

Efforts to expand access to comprehensive T1D care are currently underway in several countries across multiple regions of the world, particularly through PEN-Plus strategies to offer integrated NCD care at the district hospital level. Our findings have implications for the design of future and ongoing T1D care programs. They demonstrate that social support interventions such as food distribution, home visits, or cash transfers may be essential in LICs to ensure good T1D control given the impact of food insecurity on self-management. Individualized education of PLWT1D and their families should be viewed as a key component of T1D care to facilitate optimal self-management. While providers did note low patient literacy and numeracy to be a potential barrier to care, they explained that its impact on self-management can be mitigated through extensive, continuous patient education at clinic visits. This study as well as our similar work in Liberia demonstrate the potential benefits of diabetes self-management education, a program currently being introduced by PIH in Liberia, to facilitate knowledge, skills, and ability for T1D self-care. This approach helps patients develop problem-solving skills due to diabetes management issues they may encounter, including the barriers to care identified in this study. Given the low community awareness and underdiagnosis reported of T1D reported by providers, community level education should also be considered as part of T1D programs.

## Conclusions

In this study, we found T1D to have a significant psychosocial and economic impact to PLWT1D and their families. Overall, PLWT1D reported good knowledge and practice of self-management activities, facilitated by extensive diabetes education, provision of free insulin and supplies, home visits, and distribution of transport funds. However, patients faced significant barriers to care such as poor geographic access, food insecurity, and low literacy. Our findings more broadly demonstrate the need for additional research and programs to address such barriers to T1D care in low-resource settings like Malawi.

## Electronic supplementary material

Below is the link to the electronic supplementary material.

Supplementary Material 1

Supplementary Material 2

Supplementary Material 3

## Data Availability

The datasets used and/or analyzed during the current study are available from the corresponding author on reasonable request.

## List of Abbreviations

APZU: Abwenzi Pa Za Umoyo
BG: Blood glucose
CHW: Community health worker
IDF: International Diabetes Federation
NCD: Noncommunicable disease
NGO: Non-governmental organization
PIH: Partners in Health
PLWT1D: People living with type 1 diabetes
SSA: sub-Saharan Africa
T1D: Type 1 diabetes

## References

[1] World Health Organization & International Diabetes Federation. Definition and diagnosis of diabetes mellitus and intermediate hyperglycaemia: report of a WHO/IDF consultation. 2006.

[2] Atun R, Davies JI, Gale EAM, Bärnighausen T, Beran D, Kengne AP et al. Diabetes in sub-Saharan Africa: from clinical care to health policy. Lancet Diabetes Endocrinol. 2017 Aug;5(8):622–667. doi: 10.1016/S2213-8587(17)30181-X. Epub 2017 Jul 5. PMID: 28688818. 10.1016/S2213-8587(17)30181-X 28688818

[3] IDF Diabetes Atlas. International Diabetes Federation. 2021. https://www.diabetesatlas.org/data/en/country/119/mw.html. Accessed 8 Aug 2022.

[4] Malawi Ministry of Health. Malawi National STEPwise Survey for Non-Communicable Diseases Risk Factors 2017 Report. Lilongwe: Ministry of Health. ; 2021. https://dnhamalawi.org/wp-content/uploads/2020/12/2017-STEPS-Survey-Report-Malawi.pdf. Accessed 7 Sept 2022.

[5] Beran D, Hirsch IB, Yudkin JS (2018). Why are we failing to address the issue of access to insulin? A national and global perspective. Diabetes Care.

[6] Gupta N, Coates MM, Bekele A, Dupuy R, Fenelon DR, Gage AD (2020). Availability of equipment and medications for non-communicable diseases and injuries at public first-referral level hospitals: a cross-sectional analysis of service provision assessments in eight low-income countries. BMJ Open.

[7] Assayed AA, Muula AS, Nyirenda MJ (2014). The quality of care of diabetic patients in rural Malawi: a case of Mangochi district. Malawi Med J.

[8] 3 Axis Advisors. A global comparison of insulin prices. 2021. https://static1.squarespace.com/static/5c326d5596e76f58ee234632/t/618b3af402c03725b63672a4/1636514553353/MPZ_FINAL_2021_11_09.pdf. Accessed 28 Jul 2022.

[9] Ruderman T, Chibwe E, Boudreaux C, Ndarama E, Wroe EB, Connolly E, Bukhman G (2022). Training mid-level providers to treat severe non- communicable Diseases in Neno, Malawi through PEN-Plus Strategies. Annals of Global Health.

[10] Partners In Health: Malawi. https://www.pih.org/country/malawi. Accessed 28 Jul 2022.

[11] Wroe EB, Kalanga N, Dunbar EL, Nazimera L, Price NF, Shah A (2020). Expanding access to non-communicable disease care in rural Malawi: outcomes from a retrospective cohort in an integrated NCD-HIV model. BMJ Open.

[12] NCDI Poverty Network. : PEN-Plus. http://www.ncdipoverty.org/penplus-1. Accessed 28 Jul 2022.

[13] Adler AJ, Trujillo C, Schwartz L, Drown L, Pierre J, Noble C (2021). Experience of living with type 1 diabetes in a low-income country: a qualitative study from Liberia. BMJ Open.

[14] Michie S, Johnston M, Abraham C, Lawton R, Parker D, Walker A (2005). Making psychological theory useful for implementing evidence based practice: a consensus approach. Qual Saf Health Care.

[15] Michie S, Atkins L, West R (2014). The behaviour change wheel: a guide to designing intervention.

[16] Davies P, Walker AE, Grimshaw JM (2010). A systematic review of the use of theory in the design of guideline dissemination and implementation strategies and interpretation of the results of rigorous evaluations. Implement Sci.

[17] Adler AJ, Laar AK, Kotoh AM, Legido-Quigley H, Perel P, Lamptey P (2020). Barriers and facilitators to the implementation of a community-based hypertension improvement project in Ghana: a qualitative study of ComHIP. BMC Health Serv Res.

[18] Laar AK, Adler AJ, Kotoh AM, Legido-Quigley H, Lange IL, Perel P (2019). Health system challenges to hypertension and related non-communicable diseases prevention and treatment: perspectives from ghanaian stakeholders. BMC Health Serv Res.

[19] Dedoose. Version 8.3.35, web application for managing, analyzing, and presenting qualitative and mixed methods research data [program] 2020.

[20] Ruderman T, Ferrari G, Valeta F, Boti M, Kumwenda K, Park PH et al. Implementation of self-monitoring of blood glucose for insulin dependent diabetes patients in a rural non-communicable disease clinic in Neno, Malawi. S Afr Med J. 2022. 10.7196/SAMJ.2023.v113i2.16643 36757071

[21] Nkambule E, Msosa A, Wella K, Msiska G (2021). This disease would suit better those who have money’: insulin-treated diabetes illness experience in rural Malawi. Malawi Med J.

[22] Kratzer J (2012). Structural barriers to coping with type 1 diabetes mellitus in Ghana: experiences of diabetic youth and their families. Ghana Med J.

[23] Hapunda G, Abubakar A, van de Vijver F, Pouwer F (2015). Living with type 1 diabetes is challenging for zambian adolescents: qualitative data on stress, coping with stress and quality of care and life. BMC Endocr Disord.

