# Supplementary material for: Living with type 1 diabetes in Neno, Malawi: a qualitative study of self-management and experiences in care
Source: BMC Health Serv Res. 2023 Jun 8;23:595. doi: 10.1186/s12913-023-09519-z (PMC10248969; doi:10.1186/s12913-023-09519-z)
Supplement: Supplementary file 2 — Supplementary Material 2 [file 12913_2023_9519_MOESM2_ESM.docx]

Appendix 1: Interview guides

**T1D Qualitative Interview Guide: Patients**

| **Question** |
| --- |
| 1. How often do you attend school/work? |
| 1. How do you enjoy school? |
| 1. Can you tell me about your family structure? Who do you live with? |
| 1. Who assists you on a day to day basis with your T1D? |
| 1. How long have you had T1D? |
| 1. Pre-diagnosis, had you heard of diabetes? What did you know about it? |
| 1. Does anyone else in your family have a history of diabetes? Do any of your friends? |
| 1. When you first became ill, what prompted you to seek care? |
| 1. How were you diagnosed? (probe for symptoms, length of time before diagnosis) |
| 1. Do you use any alternative treatments other than the ones that your provider has given you? |
| 1. Can you walk me through a typical clinic visit? |
| 1. How often do you attend? |
| 1. How often do you miss appointments?   Why do you miss appointments?  How long did you wait to reschedule or attend a new appointment? |
| 1. When you go to clinic who do you normally see? (probe for type of HCP, etc, do they normally see same person, or different every time) |
| 1. Does your HCP ask about your feelings about care? |
| 1. What is your treatment schedule (probe to see if it is clear and if they have knowledge) |
| 1. How long do you usually wait to see your HCP at the clinic? |
| 1. Do you ever leave the clinic without seeing a health care provider? Can you give me an example? Were you given a reason for it? (probe for wait time, unavailable HCP, etc) |
| 1. How often do you see your pharmacist? (probe for interaction, i.e., do they just pick up drugs, or have any counselling) (if they do not see pharmacist ,skip to number 24) |
| 1. Have you received good information (counseling) from the pharmacist? |
| 1. Where is the pharmacist located (in hospital, do they have to travel)? Do you see the same pharmacist, or different ones? |
| 1. How long do you have to wait to see the pharmacist? |
| 1. How regularly do you leave without your needed medications? When this happens, what do they tell you? What do you do? Where do you get your needed medications? |
| 1. Have you ever taken a reduced dose of your medication? How often? Why did you do this? (probe for cost, stock-out, make it last longer, didn’t think they needed that much, etc) |
| 1. Do you regularly see a community health worker? If so, do you speak with them about your T1D? Can you tell me about an interaction with them? How have they helped you? How have they influenced your care? 2. How would you rate the quality of care that you receive? |
| 1. What is one thing that would improve the quality of your health care experience? |
| 1. Could you walk me through a typical day regarding your illness? (probe for if they check their blood glucose, if so how often, do they have a log book, anything else that may be part of day to day care) |
| 1. How often do you check your blood glucose? |
| 1. When do you check your blood glucose? (Probe for different times of the day, different days, etc) |
| 1. Can you tell me about a time that you did not use a glucose strip? Can you tell me about your reasons for not doing so? (probe for lack of availability of strips, or cost of strips) |
| 1. Do you check your blood glucose yourself or does someone help you? Who? |
| 1. How do you use your blood glucose reading to inform your self-management? (probe for insulin, diet, exercise, water) |
| 1. How do you know when your blood glucose is out of its range? |
| 1. How often do you experience hypoglycemic events? |
| 1. How often do you experience hyperglycemic events? |
| 1. How do you manage your hyper/hypo glycemic events? |
| 1. How often do you need help to manage your hyper/hypo glycemic event? (probe for severe) |
| 1. What proportion of your day do you spend worrying about your diabetes? |
| 1. What proportion of your day do you spend managing your diabetes? |
| 1. Do you feel like you have your condition under control? |
| 1. How often do you take insulin? Do you ever skip doses? Why? |
| 1. Who gives you injections? |
| 1. How do you store your insulin? (probe for knowledge on cold chain) |
| 1. Have you engaged with any support groups, camps or diabetes associations? What have you gained from these interactions? (probe for financial, access to HCP, psychological support, knowledge) |
| 1. Discuss how your life has changed since your diagnosis? How does it affect what you eat? What you drink? What you do on a day-to-day basis? What activities you participate in? |
| 1. Has your perception of your future changed since your diagnosis? |
| 1. Do you check your blood glucose in public? Do you have injections in public? Why or why not? |
| 1. Can you tell me about a time you tried to hide your condition? |
| 1. Do you have insurance? |
| 1. How often do **you** miss work/school because of your condition? Include clinic visits, illness, hospitalization. How often does a **family member or carer** miss work because of your condition? |
| 1. What are your typical out of pocket costs for transport? Insulin? Strips? Days off of work? |
| 1. Activity: Show cards with different readings on them. What would they do in each scenario? |

**T1D Qualitative Interview Guide: Family members**

| **Question** |
| --- |
| 1. Can you tell me about what you do for a job? |
| 1. Can you tell me about your family structure? Who lives with you in your house? |
| 1. Can you tell me about the structure of your house? |
| 1. Can you tell me about your family member who has T1D? |
| 1. How long has your family member had T1D? |
| 1. Pre-diagnosis, had you heard of diabetes? What did you know about it? |
| 1. Does anyone else in your family have a history of diabetes? Do any of your friends? |
| 1. When your family member first became ill, what prompted them to seek care? How were they diagnosed? (probe for symptoms, length of time before diagnosis) |
| 1. Do you think alternative treatments other than the ones that the provider you know has prescribed are effective? |
| 1. How often do you attend your family member’s clinic visits? |
| 1. Can you walk me through a typical clinic visit that you attend? |
| 1. How far do you have to travel to see the health care provider? |
| 1. How often does your family member miss appointments? Why do they miss appointments? How long did they wait to reschedule or attend a new appointment? |
| 1. When you go to clinic who do you normally see? (probe for type of HCP, etc, do they normally see same person, or different every time) |
| 1. Does your HCP ask about your feelings about care? |
| 1. What is your family members’ treatment schedule? (probe to see if it is clear and if they have knowledge) |
| 1. How long do you usually wait to see their HCP at the clinic? |
| 1. Do you ever leave the clinic without seeing a health care provider? Can you give me an example? Were you given a reason for it? (probe for wait time, unavailable HCP, etc) |
| 1. When attending a T1D appointment, how often do you see a pharmacist? (probe for interaction, i.e., do they just pick up drugs, or have any counselling) (if they do not see a pharmacist, skip to number 24) |
| 1. Have you received good information (counseling) from the pharmacist? |
| 1. Where is the pharmacist located (in hospital, do they have to travel)? Do you see the same pharmacist, or different ones? |
| 1. How long do you have to wait to see the pharmacist? |
| 1. How regularly do you leave without the needed medications? When this happens, what do they tell you? What do you do? Where do you get the needed medications? |
| 1. Has your family member ever taken a reduced dose of your medication to make it last longer? How often? Why (probe for cost, stock-out, etc) |
| 1. Do you regularly see a community health worker? If so, do you speak with them about your family member’s T1D? Can you tell me about an interaction with them? How have they helped you? How have they influenced your family member’s care? |
| **Treatment and self-management:** |
| 1. How do you know when your family member’s blood glucose is out of its range? |
| 1. How do you help your family member manage their hyper/hypo glycemic events? |
| 1. What proportion of your day do you spend thinking about your family member’s diabetes? |
| 1. What proportion of your day do you spend helping manage diabetes? |
| 1. Do you feel like your family member has their condition under control? |
| 1. Do you help give injections? |
| 1. How is insulin stored in your household? (probe for knowledge on cold chain) |
| 1. Are you aware of any peer support groups? |
| 1. Discuss how your life has changed since your family member’s diagnosis? How does it affect what you eat? What you drink? What you do on a day to day basis? What activities you participate in? |
| 1. Has your perception of your future changed since your family member’s diagnosis? |
| 1. How often do **you** miss work/school because of your family member’s condition? Include clinic visits, illness, hospitalization. |
| 1. What are your typical out of pocket costs for transport? Insulin? Strips? Days off of work? |
| 1. Activity: Show cards with different readings on them. What would they do? |

**Interview Guide with Health Care Professionals**

**Please remember this is a guide only** - conduct these interviews following the topic guide but allow for flexibility/

Interviews with health care professionals should focus on their expertise and the role they play in the treatment of T1D, as well as understanding their relationship with the patient, how the care is coordinated; the information they provide to the patient; and what are the areas that are needed for improvement. In addition, the interviewer should try to find out what is the health professional’s knowledge of the existent regional, national programmes to prevent and treat T1D. The interviewer should also ask questions on the use and implementation of clinical guidelines. The question will be tailored to whether the interviewee is a community worker, nurse, GP or secondary care specialist.

|  | **HEALTHCARE PROFESSIONALS’ EXPERIENCE AND TASKS** |
| --- | --- |
| 1. | Can you tell me about your role as a clinician/nurse/specialist? |
| 2. | What is your level of training? |
| 3. | How is service organized at your facility? |
| 4. | Can you describe the other health care professionals you work with? |
| 5. | How is T1D diagnosed? What are the key steps? |
| 6. | What are the key symptoms? |
| 7. | Can you tell me about the training that you have had specifically for T1D? Who provided the training? When did you receive your training? |
| 8. | How often do you have continuing medical education? How much of it is specific to T1D? |
| 9. | How often do you see patients with T1D? |
| 10. | Do you feel that your training is adequate for treating patients with T1D? |
| 11. | What other training do you think you would benefit from? |
|  | **TREATMENT** |
| 12. | How is T1D care organised at your facility? (probe for if they have clinics, individual appointments, etc) |
| 13. | Could you please explain to me the steps that a patient follows when they come to you and you suspect T1D? (probe for diagnosis/symptoms, comorbidities, severity, family history, etc) |
| 14. | What services do you provide to patients with T1D at your facility? (probe for medications, education, schedule of care) |
| 15. | How is this care coordinated with the rest of the team you work with, and with other services (secondary care, primary care)? |
| 16. | When a patient is first diagnosed what education initially is provided? |
| 17. | How often does the patient receive follow-up education? How is this education structured? |
| 18. | What are the things that you think work well? |
| 19. | How do you think you could coordinate care better? What are the biggest challenges? |
| 20. | How many patients do you typically see a day? How many have T1D? |
|  | **CLINICAL GUIDELINES** |
| 21. | What clinical guidelines on T1D exist in Malawi? How were these developed? |
| 22. | What are your views on clinical guidelines? Do you think these are useful? |
| 23. | What are some of the barriers/facilitators of implementing clinical guidelines? |
| 24. | Are clinical guidelines visible/available in your facility? |
| 25. | How often do you reference clinical guidelines? |
|  | **RELATIONSHIPS WITH PATIENTS** |
| 26. | Can you tell me about your interactions with patients? What are challenges to effective communication? |
| 27. | Overall how would you rate the literacy/numeracy in your treatment population? Do you feel it is sufficient for effective self-management? |
| 28. | What do you think are the key challenges to adherence to treatment in your own experience? |
| 29. | What areas of patient education should be improved? How could this be implemented? |
| 30. | Do you feel that you have sufficient time with each patient? Why or why not? |
| 31. | To what extent do your patients apply the knowledge that you give them? (Prompt: do they feel their patients listen to them) |
| 32. | To what degree are parents/carers/family members (when relevant) included in the consultation? |
|  | **HEALTH SYSTEM ISSUES** |
| 33. | What areas do you think work well in your system regarding T1D? |
| 34. | Do you think all patients have the same access to care? Why or why not? (Probe for distance, SES, literacy/numeracy) |
| 35. | What areas could be improved to make your work easier and more enjoyable? (Probe for: Is funding a key concern for you? Lack of physical resources? Or the lack of human resources, or the information systems, or poor facilities, or access to care?) |
| 36 | What could be done to improve the patient journey through the health system? |
| 37. | What questions do you ask about patient’s current health and self-management? (treatment) |
| 38. | What else do you screen for? (Probe for mental health questions, wellbeing, co-morbidities) |
| 39. | Who else do you recommend the patients speak with? (probe for social workers, peers support, civil society groups, pharmacists, etc) |
| 40. | What social support mechanisms are available to your patients? (probe for counselling, travel expenses, community health worker assignments, etc) |
| 41. | How do your patients get access to them? |
| 42. | Can you talk about the different referral pathways regarding how a patient may end up in your care? |
| 43. | Under what circumstances do you refer patients to other providers? |
| 44. | Are there age restrictions to care? What happens when patients age out of philanthropy programmes? (for example, after they are 18 no longer paediatric) |
| 45. | Does the level and pathways of care change as a patient ages? |
| 46. | For patients with health insurance, what aspects of care does it cover? |

**T1D Qualitative Interview Guide: Civil Society**

| **Question** |
| --- |
| 1. Can you please describe your organization? |
| 1. Where is your organization located? |
| 1. Can you please describe your role at this organization? |
| 1. How long has your organization provided services? |
| 1. Who does your organization provide services for? |
| 1. What level of training do members of your organization have? Can you talk through typical training? |
| 1. Which health care facilities do you work at in Malawi? Can you provide approximate patient numbers? |
| 1. What services does your organization provide? |
| 1. Who provides these services? (expats vs local hires) |
| 1. How are services organized at the point of care? |
| 1. How are T1D services provided compared to other services at the point of care? |
| 1. How do you work alongside other existing private or governmental organizations? |
| 1. Does your organization subsidize human resource training and education? |
| 1. Does your organization provide social support services? If so, how? |
| 1. Can you describe a case of how you supported a T1D patient? |
| 1. Who does your organization provide services for? |
| 1. What age or restrictions do you have for inclusion for your services? |
| 1. Do you have a transition plan for patients that no longer receive your services? |
| 1. Does your organization prioritize low SES patients? If so, how? |
| 1. What other at-risk groups does your organization support? |
| 1. What is the role of the individual and community in providing T1D services through your organization? |
| 1. What challenges do you see regarding patient inclusion for your organization? |
| 1. Where within the health system does your organization provide services? |
| 1. How are these sites chosen? |
| 1. How and when are patients referred to different levels of the health system? |
| 1. How does the SES of the population inform where services are provided? |
| 1. How does your organization support hard to access patients? |
| 1. Are there costs to the patient for receiving your services? |
| 1. How does your organization fund these services? |
| 1. How are consumables, namely insulin and diagnostic equipment, financed by your organization? |
| 1. How does your organizations procurement and supply chains relate to those of the public sector and/or other private organizations? |
| 1. How does your organization address sustainability of the services you provide? |
| 1. How does your organization work with the public sector? |
| 1. Does your organization have a set timeline of when you are able to provide services? |
| 1. How will the capacity your organization has developed be transitioned if/when this timeline approaches? |
| **Closing Questions** |
| 1. What are your hopes for the prevention of complications and management of T1D in Malawi? |
| 1. Before we wrap up, do you have any final thoughts that you’d like to share? |
| 1. This brings us to the end of our interview for today. Do you have any parting questions? |
