# Supplementary material for: Living with type 1 diabetes in Neno, Malawi: a qualitative study of self-management and experiences in care
Source: BMC Health Serv Res. 2023 Jun 8;23:595. doi: 10.1186/s12913-023-09519-z (PMC10248969; doi:10.1186/s12913-023-09519-z)
Supplement: Supplementary file 3 — Supplementary Material 3 [file 12913_2023_9519_MOESM3_ESM.docx]

Appendix 2

Supplementary figure 1: Coding tree
